# MiR-9 promotes the phenotypic switch of vascular smooth muscle cells by targeting KLF5

**DOI:** 10.3906/sag-1710-173

**Published:** 2019-06-18

**Authors:** Xiaochun LU, Shi-Tang MA, Bo ZHOU, Tieling LI

**Affiliations:** 1 Department of Geriatric Cardiology, Chinese People’s Liberation Army General Hospital, Beijing China; 2 Food and Drug College, Anhui Science and Technology University, Fengyang China; 3 Department of Geriatric, Affiliated Zhongda Hospital of Southeast University, Nanjing China; 4 Department of Cadre Clinic, Chinese People’s Liberation Army General Hospital, Beijing China

**Keywords:** miR-9, smooth muscle cells, proliferation, migration, KLF5

## Abstract

**Background/aim:**

Diabetic vascular smooth muscle cells (VSMCs) are characterized by increased proliferation and migration. Small noncoding microRNAs (miRNAs) have been considered critical modulators of the VSMC phenotypic switch after an environmental stimulus. However, microRNA in high glucose-induced proinflammation and its atherogenic effect is still ambiguous.

**Materials and methods:**

The technique of qRT-PCR was used to examine the expression of miR-9 in VSMCs. The downstream signaling protein relative to miR-9 regulation, Krüppel-like factor 5, and some marker genes of contractile VSMCs were analyzed by western blotting and qRT-PCR. Luciferase reporter assay was used to detect the expression of KLF5, which is regulated by miR-9. To examine the function of a miR-9 inhibitor in VSMC proliferation and migration, VSMC proliferation and migration assays were performed.

**Results:**

Reduced transcriptional levels of miR-9 and expression of specific genes of contractile VSMCs were observed in the SMC cell line C-12511 treated with high glucose and SMCs, which were isolated from db/db mice. Moreover, the activity of KLF5 3′-UTR was dramatically reduced by a miR-9 mimic and increased by a miR-9 inhibitor. The proliferation and migration of SMCs were reduced by the miR-9 mimic.

**Conclusion:**

miR-9 inhibits the proliferation and migration of SMC by targeting KLF5 in db/db mice.

## 1. Introduction

Cardiovascular disease, especially atherosclerosis that may result in mortality, is the leading complication in patients with diabetes mellitus (1). The common pathological characteristics of coronary atherosclerosis and subsequent fatal or nonfatal myocardial infarction result in the VSMCs regaining their ability to proliferate and migrate. It is widely accepted that the phenotypic conversion of VSMCs (vascular smooth muscle cells) might be the critical cause of atherosclerosis lesion formation and restenosis after angioplasty or bypass (2,3). The physiological contractile phenotype of VSMC can undergo a phenotypic change to the synthetic phenotype in response to pathological environmental stimuli, such as platelet-derived growth factors, hyperglycemia, and balloon injury. The synthetic VSMCs regained the abilities of migration and proliferation but resulted in the loss of contractility and subsequent vessel occlusion.

Previous studies have shown that the proliferation and hyperglycemia-induced inflammatory responses are higher in VSMCs from a diabetic model than in those from a healthy control (4,5), suggestive of abnormal regulation of differentiation processes in diabetic VSMCs. Some key pathological factors associated with diabetes, including high glucose (HG), advanced glycation end products, growth factors, and oxidized lipids, would promote VSMC dysfunction in diabetic (db/db) mice. Nevertheless, little is known about the mechanism of abnormal VSMC proliferation and differentiation in patients with diabetes. Therefore, the potential ability of VSMCs to dedifferentiate in response to environmental cues such as growth factors, inflammatory cytokines, and high glucose-induced culture conditions should be further elucidated to clarify the pathogenic mechanism of vascular diseases relative to diabetes (6). 

KLF5, a member of the family of Kruppel-like factors, belongs to a group of transcription factors containing zinc finger domains, and serves as a transcriptional activator or repressor, regulating a variety of physiological processes, including differentiation, development, and proliferation (7,8). However, KLF5 is also modulated by a variety of biological mechanisms, including microRNA, and other transcriptional factors (9–11). Previous studies have demonstrated that KLF5 might contribute to VSMC dedifferentiation and synthetic phenotype change in balloon-injured artery and platelet-derived growth factor (PDGF)-stimulated VSMCs (9). The study revealed the conceivable regulation mechanism of VSMC dedifferentiation in pathological conditions relative to atherosclerosis formation, including stimulation by growth factors and vascular endothelial injury.

It has been revealed that by regulating expression of several transcription factors in various pathological conditions, microRNAs influence VSMC differentiation and proliferation (4). The microRNAs regulate their target genes by inducing translational repression or mRNA degradation. It has been reported that miR-663 might promote neointima formation and the VSMC phenotypic switch by targeting JunB/myosin light chain 9 expression (12). It has also been suggested that several other miRNAs, such as miR-26a and miR-22, also acted upon VSMC differentiation in the given pathological conditions (13,14). However, the regulation of microRNA is quite complicated and distinct in different pathological conditions. The expression of multiple genes might be regulated by microRNAs via their binding to mRNA targets; the specific mRNA is also regulated by a variety of microRNAs based on the accessible sequence of 3′-UTR region. MiR-9 has also been proven to be related to the protective effect in VSMCs after balloon injury via degeneration of platelet-derived growth factor receptor (PDGFR) (15). However, miR-9 promoted the proliferation of pulmonary artery smooth muscle cells (PASMCs) after hypoxia stimulation, which demonstrated that miR-9 has different effects in distinct pathological conditions (16). Therefore, a continuous in-depth study is essential to understand this comprehensive regulation mechanism.

In the present study, the differential expression of miR-9 in VSMCs from db/db mice and db/+ controls was found, in reference to the sequencing profiles of small RNAs in VSMCs from db/db mice. This highly conserved miRNA promotes microglial activation and inflammatory responses by targeting MCPIP1 and mediating NF-κB signaling (17), but its function in VSMCs and diabetes complications is unknown. By bioinformatic analysis, we then recognized that KLF5 is the target gene of miR-9; KLF5 downregulates the expression of myocardin. The downstream molecule myocardin might be responsible for the phenotypic conversion of VSMCs, which influences the dysregulation of miR-9 in promoting VSMC dedifferentiation.

## 2. Materials and methods

### 2.1. Cell Culture

Human aortic SMCs (C-12511, Promocell, Karlsruhe, Germany) and HEK293 cells were cultured in Dulbecco’s modified Eagle’s medium (DMEM) (provided by Gibco Life Technologies, Gaithersburg, MD, USA), which contained 100 units/mL of penicillin, 100 μg/mL of streptomycin, and 10% FBS, and was low in glucose in a humidified incubator at 37 °C with 5% CO2. 

### 2.2. Separation and culture of VSMC in mice

The study was approved by our Institutional Animal Ethics Committee. VSMCs were derived from the thoracic aorta of male db/db mice aged 10–12 weeks (B6.BKS(D)-Leprdb/Nju) and nondiabetic db/+ mice (age-matched) provided by the National Resource Center of Model Mice (NRCMM) and Model Animal Research Center of Nanjing University. The protocol was performed as described previously (18). After the mice were killed, the thoracic aortas were harvested. The adipose tissues and connective tissues of the aorta were removed, and the aortas were then incubated with DMEM/F12 + 0.2% BSA which contained 0.3 mg/mL elastase (provided by Sigma, St. Louis, MO, USA) and 1 mg/mL collagenase B (provided by Roche, Basel, Switzerland) at 37 °C for 8–10 min. It was further digested at 37 °C for 1 h following the removal of the adventitia. The tissues were then cut into pieces and centrifuged at 200 g at room temperature for 5 min. After washing, the VSMC pellets were plated in DMEM-F/12, which was supplemented with 100 μg/mL of streptomycin, 100 units/mL of penicillin, and 20% FBS. The purity of VSMCs reached over 80% based on morphology under light microscope and immunofluorescent staining for α-smooth muscle actin. VSMCs were then cultured in DMEM/F12 medium, which was supplemented with streptomycin, penicillin, and 10% FBS. VSMCs from passages 4–6 were used for the following experiments.

### 2.3. Oligo transfection, overexpression, or knockdown of KLF5 and miR-9 in cultured VSMCs

The expression of miR-9 was decreased by the miR-9 inhibitor and increased by a miR-9 mimic (Thermo Fisher Scientific, Waltham, MA, USA). KLF5 and myocardin knockdown was performed using their respective siRNA (si-KLF5, 100 nM, Thermo Fisher Scientific) and siRNA-MYOCD (80 nM, Thermo Fisher Scientific). An adenovirus which expressed KLF5 (Ad-KLF5) was used for the KLF5 upregulation assay. Cells were transfected using LipofectamineTM RNAiMAX (for oligonucleotides) or Lipofectamine® 2000 according to the manufacturer’s protocol. Vehicle, oligo controls (Thermo Fisher Scientific), siRNA control (Thermo Fisher Scientific), and adenovirus control (Ad-GFP) were also used.

### 2.4. Construction of the adenovirus vector 

The construction of adenoviruses encoding mouse- or human-KLF5 (Ad-KLF5), mice- or human-myocardin (Ad-MYO), and GFP (Ad-GFP) was authorized by Invitrogen. When VSMCs derived from mice aortas or C-12511 cells attained 80% confluence, the cells were infected by KLF5 adenovirus or control virus at a concentration of 106/50 μL cultured medium for 24 h (MOI = 200~300) and treated for 12 h. Cells were then collected and lysed for subsequent assays. 

### 2.5. RNA analysis by quantitative real-time PCR (qRT-PCR)

Total RNA was extracted by miRNAeasy mini kits (217004, Qiagen, Hilden Germany) in accordance with the manufacturer-supplied protocols. Reverse transcription and qPCR were employed to analyze gene expression and miRNA transcription. Following the reverse transcription reactions, qPCR for miRNAs was carried out using a miScript SYBR Green PCR Kit (provided by Qiagen); the mRNA expression was analyzed using SYBR green PCR master mix (provided by Applied Biosystems, Foster City, CA, USA). All qPCR reactions were performed in double wells on 7300 or 7500 qPCR equipment (Applied Biosystems) in 20 µL total reaction volume. The 2-∆∆ Ct method was used to analyze qPCR data. The transcriptional level was normalized to the expressions of U6 and β-actin for mRNAs and miRNAs, respectively. Fold-changes with respect to the values of the db/+ or control samples were used to describe the results. The sequences of the primers used are shown as following: Calponin F: 5′-ATC GTT GTA CAA TGGT-3′, R: 5′-TAC CCC ATT TGG CCA TTT AAA AAT CT-3′; SMA F: 5′-TAC CTC ATT GGT TAC TCC AGGT-3′, R: 5′-CTT AGT TAC CTG TTT AGA TTG CC-3′; SM-22α F: 5′-GCC GTT ACC GTT GAT CCA GTT TT-3′, R: 5′-GTT TTC CTA GTC CTT CCG AT-3′; KLF5 F: 5′-GTC TAG TTC AGT CAG TTC AGT TGCA-3′, R: 5′-GTC CCC AGC CAC CGT AAA AAA TGAC-3′; GAPDH F: 5′-TTT TGG GCT CGG TGA TTA CGA CCC AAA-3′, R: 5′-TGA TGA TTA GTT CAT ACC ACA TGAC-3′; Myocardin F: 5′-TGT CAT GCC ATG GCC CCC ATA GTT GGG TA-3′, R: 5′-TGT ACC ATC CAT TTT TTG TGT GAAA-3′.

### 2.6. Western blot analysis

Proteins from the VSMCs of animals and cell lines were extracted using lysis buffer (Boshide, Wuhan, China). After equal amounts of proteins were separated by SDS-PAGE, they were transferred onto a PVDF membrane (Millipore, Burlington, MA, USA). They were blocked with 5% milk in TTBS for 2 h at 37 °C, and incubated overnight at 4 °C with the following primary antibodies: anti-KLF5 (1:500, ab2433, Abcam), anti-SMA (1:1000, ab6594, Abcam), anticalponin (1:1000, ab32360, Abcam), anticalponin (1:1000, ab46794, Abcam), antiSM22α (1:1000, ab14106, Abcam), antimyocardin (1:1500, ab203614, Abcam), and antiGAPDH (1:1000, sc-47778, Santa Cruz Biotechnology, Dallas, TX, USA). The membranes were washed and incubated with the secondary antibodies for 1 h at 25 °C. The gray value of the protein bands was detected by ECL Fuazon Fx (Vilber Lourmat). 

### 2.7. Luciferase assay

For reporter assays, 3′-UTRs sequences of mice KLF5 and human KLF5 were amplified using specific PCR and cloned into psiCHECK vectors (provided by Promega, Madison, WI, USA), which were then placed downstream of the luciferase reporter gene, Firefly and Renilla, based on the standard procedures. DNA sequencing was used for the verification of the cloned inserts. Using nucleofection, the vectors carrying a 3′-UTR fragment of KLF5 mRNA, which contained the luciferase reporter gene and putative miR-9 binding sequence, were transfected into HEK293 cells with either oligo controls, miR-9 mimic, or a miR-9 mimic inhibitor. The psiCHECK vectors with wild type and mutational 3’UTR fragment of KLF5 were transfected into the 293HEK cells. Then the dual luciferase assays were performed to identify the specific binding site of miR-9 to 3’UTR fragment of KLF5. Two days later, the HEK293 cells were subjected to lysis, and dual luciferase assay kits (provided by Promega, Madison, WI, USA) were used to examine Firefly luciferase and Renilla activity. Firefly luciferase activity was used for normalization of the results, and fold-change over the control samples was employed to describe the results.

### 2.8. Proliferation and migration of VSMCs

Proliferation of VSMCs in vitro was determined with a Vybrant MTT Cell Proliferation Assay Kit (provided by Invitrogen, Carlsbad, CA, USA), according to the protocols provided by the manufacturer. VSMC migration assays were performed as described previously (19). SMCs were incubated in serum-free medium for 2 days. A linear wound was then gently made at the center of the cell monolayer using a 200 μL tip; the monolayer was then subjected to stimulation with high glucose and dynamic observation for 24 h. An Olympus IX 71 microscope was used to capture images. Furthermore, a colorimetric BrdU Cell Proliferation Assay (Roche, Basel, Switzerland) was used to measure cell proliferation ability. 1.0 × 104 C12511 or VSMCs were cultivated in a 96-well plate. After 24 h, serum containing medium was removed and replaced with DMEM/F12 growth medium with 1% FBS. Cells were then stimulated with 20 ng/mL PDGF-BB for 18 h. BrdU labeling, cell fixation, BrdU antibody conjugation, and color development were performed. Signal detection was performed on a SpectraMax (Roche) ELISA plate reader.

### 2.9. Statistical analysis

SPSS 20.0 software was employed for statistical analysis. The mean ± standard deviation (SD) of 3 independent experiments was used to describe the data. The difference between the 2 groups was analyzed using an unpaired t-test. Comparisons of more than 2 groups were performed with one-way analysis of variance (ANOVA). P values < 0.05 were considered statistically significant.

## 3. Results

### 3.1. Expression of miR-9 and KLF5 in VSMCs from db/db mice and high glucose-induced C-12511 cells

**Figure 1:**
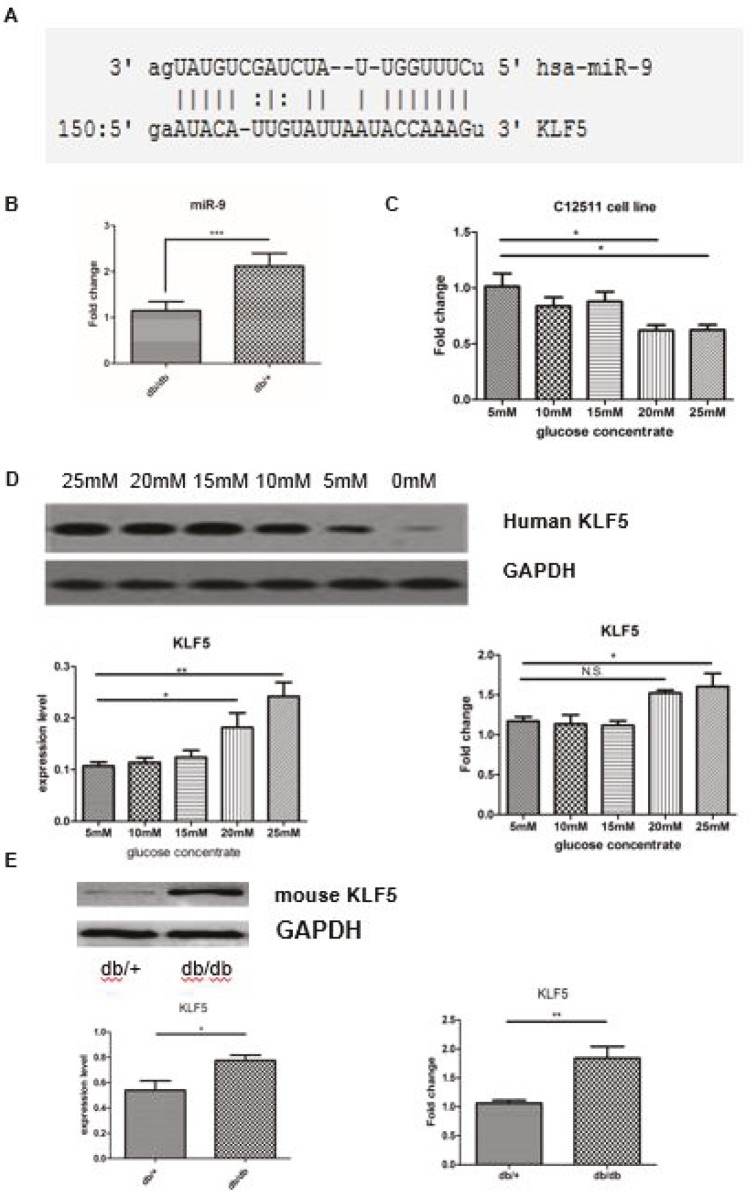
High glucose cultivation induces miR-9 downregulation and the increased expression of KLF5. A. The 3′-UTR region of KLF5 contains the binding site for miR-9. The target binding site is indicated in red. B. The decreased transcription level of miR-9 after induction with high glucose concentrations (over 20 mM) in VSMCs isolated from diabetic model animals; this was detected by performing qRT-PCR. C. The transcription level of mature miR-9 decreased after induction with high glucose concentrations (over 20 mM) in C-12511 cells; this was detected by performing qRT-PCR. D and E. Western blot and qRT-PCR for detection of KLF5 expression in C-12511 cells and VSMCs from the diabetic model. The expression of KLF5 significantly increased in C-12511 cells treated with high glucose in a dose-dependent manner (D), and in VSMCs from the diabetic model animals and control (E). The expression level was calculated by the ratio of the KLF5 to GAPDH or β-actin. Data are presented as mean ± SD; *P < 0.05 and **P < 0.01 by Student’s t-test. All experiments were replicated at least 3 times.

We identified the downregulation of miR-9 in diabetic VSMCs by reference to the profile of diabetes-induced miRNA (17); based on a bioinformatic analysis (microRNA online predictive website microRNA.org), we presumed that KLF5 could be the target gene of miR-9 (mirSVR = –1.1778, PhastCons score = 0.5217) (Figure 1A). A previous study demonstrated that miR-9 played an important role in modulating neurogenesis and axonal extension, as well as microglial inflammatory responses (17,20), but its role in the regulation of VSMC phenotypic conversion after high glucose stimulation was still unknown. To determine the dose–response relationship between KLF5 expression and glucose concentration, we performed an experiment in which C-12511 cells were treated with various concentrations of glucose (range: 5–25 mM) for 12 h; the total RNA and protein was extracted from these cells. The transcription levels of miR-9 in the VSMCs isolated from db/db and db/+ mice were measured by qPCR. We found a significantly reduced expression of miR-9 in db/db mice compared to db/+ mice (supplementary Figure 1). The VSMCs isolated from the db/+ control mice can also be used to extract the total RNA and protein for performing qPCR and Western blotting. As shown in Figures 1B and 1C, when compared with the control animals and cell group cultivated with low glucose, the expression of miR-9 notably decreased in both the VSMCs from control animals and the C-12511 cells treated with a concentration of glucose over 20 mM. The upregulation of KLF5 was also observed in C-12511 cells in a dose-dependent manner (Figure 1D); KLF5 expression was significantly increased in VSMCs from db/db mice, when compared to those of the control animals (Figure 1E). 

### 3.2. MiR-9 regulated the expression of KLF5 in VSMCs

**Figure 2:**
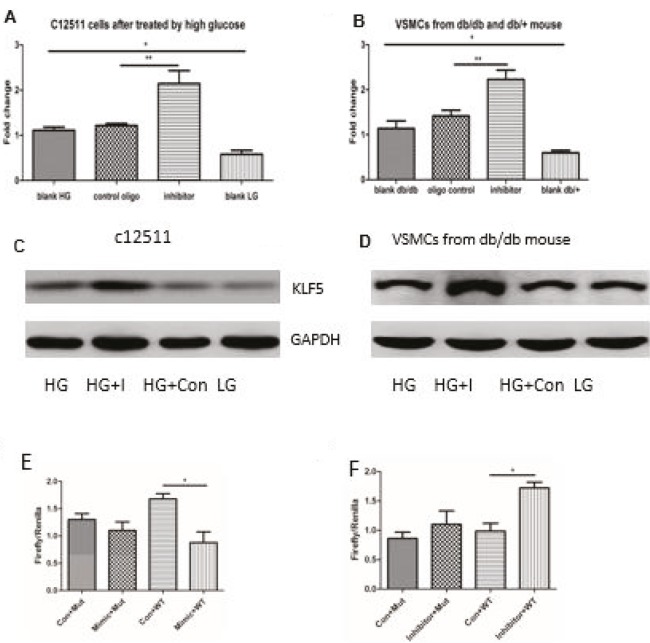
miR-9 regulated the expression of KLF5, and the miR-9 mimic attenuated the expression of KLF5 in VSMCs. A and B. Validation of miR-9 targets in VSMCs. VSMCs were transfected with miR-9 inhibitor and control oligo by nucleofection. 48 h after transfection, the expression of KLF5 was analyzed with qRT-PCR. HG: high glucose, LG: low glucose. The results showed that the expression of KLF5 was positively regulated by the miR-9 inhibitor in both C-12511 cells and VSMCs from db/db mice. C and D. The representative figures of immunoblot analysis show the expression of KLF5 in C-12511 and VSMCs isolated from db/db mice under different conditions. HG: high glucose, LG: low glucose, HG + I: high glucose + inhibitor, HG + con: high glucose + control oligos. E and F. The influence of miR-9 mimics (E) or inhibitors (F) on luciferase activity after the vectors with mutated and wild type 3’ UTR fragments of KLF5 were transfected into the HEK293 cells. Con + WT: control oligos + wild type 3’ UTR fragment, Con + Mut: control oligos + mutational 3’ UTR fragment. The results shown are representatives of 3 independent experiments. Data are presented as mean ± SD; *P < 0.05 vs. control oligo and **P < 0.01 by Student’s t-test. N = 5.

KLF5 is a suppressive transcriptional factor functioning through the interaction with the carboxy-terminal–binding protein; it promotes dedifferentiation and phenotypic conversion to synthetic VSMCs after induction by PDGF and TNF-α (9,10). To investigate the role of reduced miR-9 in vitro after high glucose stimulation-mediated KLF5 upregulation, VSMCs were transfected with a miR-9 mimic inhibitor or control before high-glucose stimulation. We examined the remaining levels of miR-9 after treatment with miR-9 inhibitors, and then we measured the enhanced levels after induction of miR-9 mimics (supplementary Figure 1). As expected, miR-9 inhibitors further promoted the expression of KLF5 in C-12511 cells after high glucose stimulation, and in VSMCs from db/db mice by RT-PCR (Figures 2A and 2B). It was also revealed that KLF5 protein expression was markedly increased in miR-9 inhibitor-treated VSMCs after high glucose stimulation in the immunoblot assays (Figures 2C and 2D). To explore whether miR-9 could bind to the 3′-UTR region of KLF5 mRNA and directly inhibit its expression, the luciferase assay was carried out in the HEK293 cells, in which the 3′-UTR fragment of KLF5 mRNA was used to construct the psiCHECK vector. As expected, only the miR-9 mimic inhibited luciferase activity. The miR-9 inhibitor was cotransfected into the HEK293 cells with the plasmid containing the 3′-UTR region of KLF mRNA, increasing the luciferase activity compared to the control inhibitor oligonucleotide (Figures 2E–2F). It was revealed that miR-9 can directly bind to KLF5 and inhibit its expression, which further elucidated the regulatory function of miR-9 in high glucose-mediated KLF5 expression in VSMCs. With the luciferase assay, we showed that miR-9 downregulated KLF5 expression in C-12511 cells by high glucose stimulation and in VSMCs from db/db mice by targeting the 3′-UTR region of KLF5 mRNA.

### 3.3. MiR-9 alleviates the phenotypic conversion of high glucose-induced VSMCs by modulating the expression of KLF5 and myocardin

**Figure 3:**
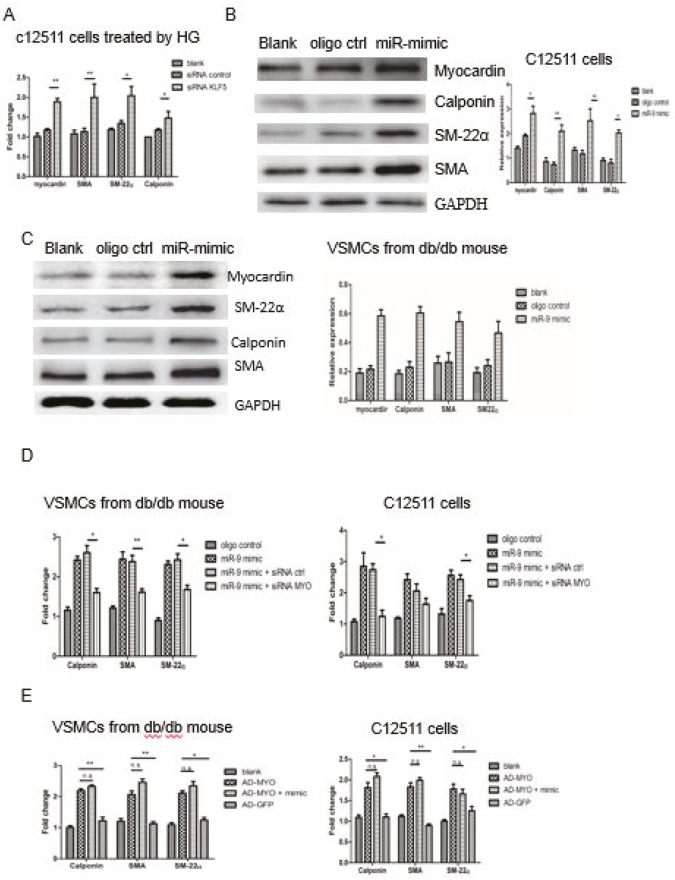
MiR-9 regulated the phenotypic conversion of high-glucose–induced VSMCs. A. 18 h after the C-12511 cells were treated with high-glucose medium and transfected with siRNA-KLF5 or control, the expression of contractile marker genes was detected by qRTPCR. B and C. 24 h after VSMCs from the db/db mice and C-12511 cells were treated with high-glucose medium and transfected with miR-9 mimic or control, the expression of myocardin and contractile marker genes was detected by Western blot. The representative immunoblots of SMA, SM22α, and calponin are shown; the relative expressions were used to demonstrate the expression change of the marker genes after the modulation of the miR mimic. D. The transcriptional level of contractile marker genes after the knockout of myocardin by transfection with siRNA MYOCDE. The reduced expression after knockout of myocardin, as expected in both C-12511 cells and VSMCs from db/db mice. No significant difference in SM22α expression was observed in C-12511 cells. E. The transcriptional level of contractile marker genes after overexpression of myocardin by transfection with AD-MYO. The expression of these genes increased after transfection with AD-MYO, independent of the induction of miR-9 mimic. Data are presented as mean ± SD; *P < 0.05 vs. control oligo and **P < 0.01 by Student’s t-test. The results shown are representative of 3 independent experiments.

To clear the availabilities of the reference gene in our study, we selected another reference, PPIA, to assess the expression stabilities in qPCR. No significant difference was found regarding the expression of b-actin among the groups treated with different oligonucleotides and adenovirus vectors in C12511 cells (Supplementary Figure 2A). In addition, we found the expression trend among different groups of KLF5 was consistent when b-actin was regarded as the housekeeping gene (Supplementary Figure 2B). A molecule present downstream of KLF5, myocardin, is considered a key modulator for VSMC differentiation marker genes (10). A previous study has demonstrated that KLF5 negatively regulates myocardin (21). To verify this in C-12511 cells after high glucose stimulation and in VSMCs from diabetic model animals, we analyzed the expression of myocardin by qPCR after transfecting the cells with siRNA-KLF5. The expression of myocardin and the marker gene of the contractile phenotype was found to be significantly increased in C-12511 cells (Figure 3A). To define the relationship between the miR-9 downregulation and phenotypic conversion, we examined the expression of contractile marker genes. As shown in Figure 3 and supplementary Figure 2, the expression of marker genes of the contractile phenotype such as SMA, SM22α, and calponin was significantly higher in C-12511 cells (Figure 3B and supplementary Figure 3A) incubated with high-glucose culture medium, and in VSMCs from db/db mice (Figure 3C and supplementary Figure 3B) after transfection with miR-9 mimic compared to those transfected with the control oligonucleotide, which suggests that the increased expression of myocardin and reduced expression of KLF5 due to the induction of the miR-9 mimic might contribute to the alleviation of phenotypic change. Furthermore, the expression of some marker genes of the contractile phenotype was lower after cotransfection with the miR-9 mimic and siRNA-MYOCD than with the miR-9 mimic and siRNA control (Figure 3D). On the other hand, the increased expression of these marker genes was independent of the induction of the miR-9 mimic after transfection with Ad-MYOCD (Figure 3E). The results elucidated that miR-9 abrogated the dedifferentiation and phenotypic conversion to synthetic VSMCs by suppressing KLF5 inhibition and the expression of myocardin.

### 3.4. MiR-9 mimic and downregulation of KLF attenuated the migration and proliferation of VSMCs induced by high glucose

**Figure 4:**
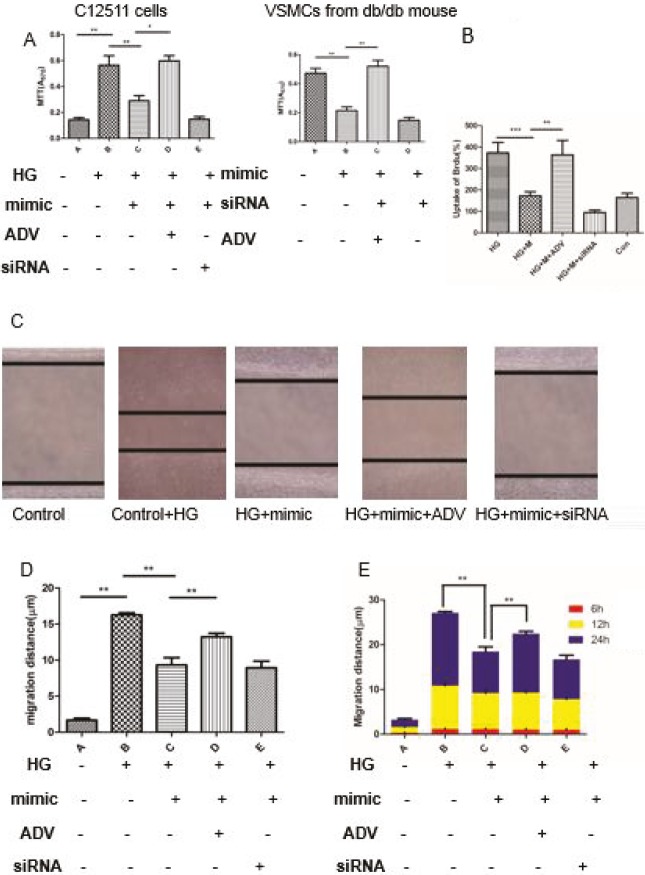
Effects of miR-9 and KLF5 on proliferation and migration in vascular smooth muscle cells (VSMCs). A and B. Cell proliferation was assessed by the MTT and Brdu cell proliferation assays. Experiments were performed in both C-12511 cells after cultivation in high glucose and VSMCs from db/db mice. C. VSMC migration was determined by a standard wound healing assay. D and E. The quantitative statistical analysis of migration distance in different groups after 24 h and migration distance at different time points (6 h, 12 h, and 24 h). HG: high glucose, mimic: miR-9 mimic, siRNA: KLF5-siRNA, ADV: adenovirus vector coding KLF5 gene. Data are presented as mean ± SD; *P < 0.05 vs. control oligo and **P < 0.01 by Student’s t-test. All of the experiments were replicated 3 times.

The function of miR-9 in the proliferation and migration of VSMCs was investigated. The cell proliferation of C-12511 cells and VSMCs were stimulated by high glucose (Figure 4A). In addition, the results of the Brdu cell proliferation assay are presented in Figure 4B, which show that the miR-9 eliminates proliferation of VSMCs when compared with control oligonucleotide. Migration ability was evaluated by the wound healing assays and the results indicated that the migration of VSMC induced by high glucose was reversed by the miR-9 mimic (Figures 4C and 4D). The migration distance at different time points is presented in Figure 4E. More importantly, Ad-KLF5 and siRNA-KLF5 contributed to a difference in the proliferative and migration ability of VSMCs by modulating the expression of KLF5 after high glucose exposure. The application of ADV-KLF5 promoted proliferation and migration, but siRNA-KLF5 had the opposite effect on biological behavior, which demonstrates that the downregulation of KLF5 via siRNA or miR-9 has a negative influence on dedifferentiation and phenotypic conversion.

## 4. Discussion

The phenotypic conversion from the physiological contractile type to the synthetic type after various kinds of extracellular stimuli and pathological conditions is observed in many vascular diseases, especially atherosclerosis, restenosis, and vascular aging (22). The terminal differentiated VMSCs are characterized by vascular smooth muscle contraction induced by AngII (23). In some conditions stimulated by pathological factors, such as inflammatory cytokines, and some growth factors, the differentiated VSMCs change to the synthetic type, with the decreased expression of some marker genes and the recovery of the proliferative and migration abilities (22,24). The dedifferentiated processes are regulated by a complicated mechanism involving transcriptional factors and noncoding small RNA. The synthetic VSMCs cannot regain the ability to proliferate but can secrete extracellular matrix and vasoactive substances, which results in intimal hyperplasia and vascular stenosis. In atherosclerotic lesions, the synthetics migrate to the intima and media layer regions and ingest the excessive liquid, which forms the foam cells (2,3). Therefore, an investigation of the regulation mechanism of the phenotypic changes of VSMCs is crucial for understanding the role of VSMC dysfunction in vascular hyperplasia lesions, and to identify the potential molecular target which could reverse the dedifferentiation process.

In a previous study, PDGF, which is commonly known as an important growth factor that results in VSMC phenotypic conversion, promoted vascular intima hyperplasia and VSMC proliferation after vascular endothelium damage. Yunhui Cheng et al. have demonstrated that miR-145 decreased after PDGF stimulation; it modulated the VSMC phenotype in cultured cells by upregulating KLF5 (9). It has been proven that microRNA-663 plays a crucial role in controlling human aortic smooth muscle cell proliferation and neointima information after PDGF-BB induction by targeting the degradation of JunB and Myl9 (12). These facts demonstrate that the downregulation of microRNA after pathological stimuli can control phenotypic changes by targeting genes which influence the migration and proliferation of VSMCs. Additionally, some transcriptional factors are also involved in the dedifferentiation and phenotype conversion. 

Numerous studies have identified the role of microRNA in various conditions. However, the regulation of VSMC phenotypic conversion in diabetic conditions is still ambiguous. Liang et al. have revealed the essential role of Pin1 in type 2 diabetes via STAT3 signaling and mitochondria-dependent pathways in restenosis (25). They found that Pin1 enhanced the migration of VSMCs and stimulated neointima hyperplasia after vascular injury by boosting VSMC proliferation. The study provided evidence that the modulation of the VSMC phenotype in diabetic conditions might be an important factor contributing to susceptibility to vascular diseases in patients with diabetes. According to a previous study, a diabetic condition indeed results in the different profile of microRNA, of which the most abundant expression is miR-504, in db/db VSMCs. It was reported that miR-504 and the gene Fgf13 in db/db VSMCs were upregulated when compared to those in db/+VSMCs, which promoted VSMC dysfunction by regulating inflammatory responses and growth factor signaling related to diabetic vascular complications. The target genes of miR-504 are Grb10 and Egr2, and the expression of inflammatory genes in VSMCs is promoted by the inhibition of Erg2, which also promotes proliferation and migration of VSMCs (22). Ham et al. (15) found that miR-9 was targeted to degenerate platelet-derived growth factor receptors (PDGFR) and protected the VSMCs from balloon injury. But whether miR-9 inhibited proliferation and migration of VSMCs is still unknown. 

In the present study, we identified another microRNA, miR-9, which decreased in VSMCs from db/db mice, with reference to the transcriptional profile of microRNA induced by diabetes from the previous study. The interaction of miR-9 with the target gene was examined using bioinformatic analysis. We found that KLF5, a key molecular switch controlling the phenotypic change by regulating marker genes such as SMA, calponin, and SM22α, is the potential downstream target gene of miR-9. We then confirmed the miR-9 transcriptional levels both in vivo and in vitro, and detected the expression of putative target genes in VSMCs from db/db mice and C-12511 cells cultivated under high-glucose conditions. As expected, the downregulation of miR-9 and the increased expression of KLF5 were observed. According to a previous study, the increased expression of KLF5 would attenuate the expression of marker genes, such as SMA, calponin, and SM22α, and promote the dedifferentiation of VSMCs. The direct effect of miR-9 on KLF5 expression was analyzed using the luciferase reporter assay. After confirmation of its interaction with KLF5, we hoped to understand whether the miR-9 inhibitor could reverse the phenotypic change characteristic of reduced expression of marker genes and the ability of VSMCs to migrate and proliferate. The results showed that the inhibitor significantly reversed the expression of marker genes. In the phenotype investigation, our study provided preliminary data about the proliferation and migration related phenotype, which suggested that the miR-9 mimic partially inhibited the growth and mobility of the VSMCs in environments of high glucose stimulation. Unfortunately, we did not perform the flow cytometry assay to evaluate the cell proliferation and we only observed the increased uptake of Brdu according to the Brdu staining. Therefore, we could not completely verify the decreased proliferative activity. The evaluation of migration abilities should also be further assessed with more experiments, such as transwell assay, to demonstrate the conclusion more precisely. To further explore the downstream mechanism, we explored some functional experiments and we concluded that myocardin is another key molecule in controlling the phenotypic conversion due to the negative influence of KLF5 and its ability to promote differentiation from the bone marrow stem cells to VSMCs. Moreover, a previous study relative to myocardin/tmem16a signaling suggested that myocardin promoted TMEM16A expression by binding to SRF and activating the promoter of TMEM16A in human aortic smooth muscle cells (HASMCs), which inhibited angiotensin II-induced proliferation in smooth muscle cells (26). Furthermore, the reduced expression of myocardin, which is negatively regulated by the overexpression of STAT3 after treatment with VEGF, would suppress the myocardin-induced upregulation of VSMC contractile phenotype-specific genes (27). These studies consistently showed myocardin to be a negative regulator for growth and phenotypic conversion. Therefore, we inferred that myocardin plays a role in the inhibition of growth induced by some pathological extracellular stimuli and contractile VSMC differentiation. This notion was then identified by the overexpression and knockout of myocardin via transfection with Ad-myocardin or siRNA-MYOCD. It was found that the positive effect of the miR-9 inhibitor on VSMC differentiation was dependent on the presence of the appropriate expression of myocardin, because the regulation of miR-9 would be absent if the expression of myocardin was silenced by siRNA-MYOCD.

In summary, our results demonstrate a novel regulation mechanism involved in the phenotypic conversion from contractile to synthetic VSMCs after high-glucose stimulus or under diabetic conditions. We discovered a potential target that could be considered a new application in the reversion of dedifferentiation in diabetic VSMCs by understanding the role of the miR-9/KLF5/myocardin axis in the regulation of phenotypic conversion.
